# The need for environmentally realistic studies on the health effects of terrestrial microplastics

**DOI:** 10.1186/s43591-023-00059-1

**Published:** 2023-05-22

**Authors:** C. Lauren Mills, Joy Savanagouder, Marcia de Almeida Monteiro Melo Ferraz, Michael J. Noonan

**Affiliations:** 1 Department of Biology, The Irving K. Barber Faculty of Science, The University of British Columbia, Okanagan Campus, Kelowna, BC Canada; 2 Clinic of Ruminants, Faculty of Veterinary Medicine, Ludwig-Maximilians University of Munich, Sonnenstr. 16, Oberschleissheim, 85764 Germany

**Keywords:** Microplastics, Terrestrial, Health, Biomimetic, Mouse, Rat

## Abstract

Plastic pollution is now so widespread that microplastics are regularly detected in biological samples surveyed for their presence. Despite their pervasiveness, very little is known about the effects of microplastics on the health of terrestrial vertebrates. While emerging studies are showing that microplastics represent a potentially serious threat to animal health, data have been limited to in vivo studies on laboratory rodents that were force fed plastics. The extent to which these studies are representative of the conditions that animals and humans might actually experience in the real world is largely unknown. Here, we review 114 papers from the peer-reviewed literature in order to understand how the concentrations and types of microplastics being administered to rodents in lab studies compare to those found in terrestrial soils. From 73 in vivo lab studies, and 41 soil studies, we found that lab studies have heretofore fed rodents microplastics at concentrations that were hundreds of thousands of times greater than they would be exposed to in nature. Furthermore, health effects have been studied for only 20% of the microplastic polymers that are known to occur in soils. Plastic pollution is arguably one of the most pressing ecological and public health issues of our time, yet existing lab-based research on the health effects of terrestrial microplastics does not reflect the conditions that free-ranging vertebrates are actually experiencing. Going forward, performing more true-to-life research will be of the utmost importance to fully understand the impacts of microplastics and maintain the public’s faith in the scientific process.

**Graphical Abstract:**

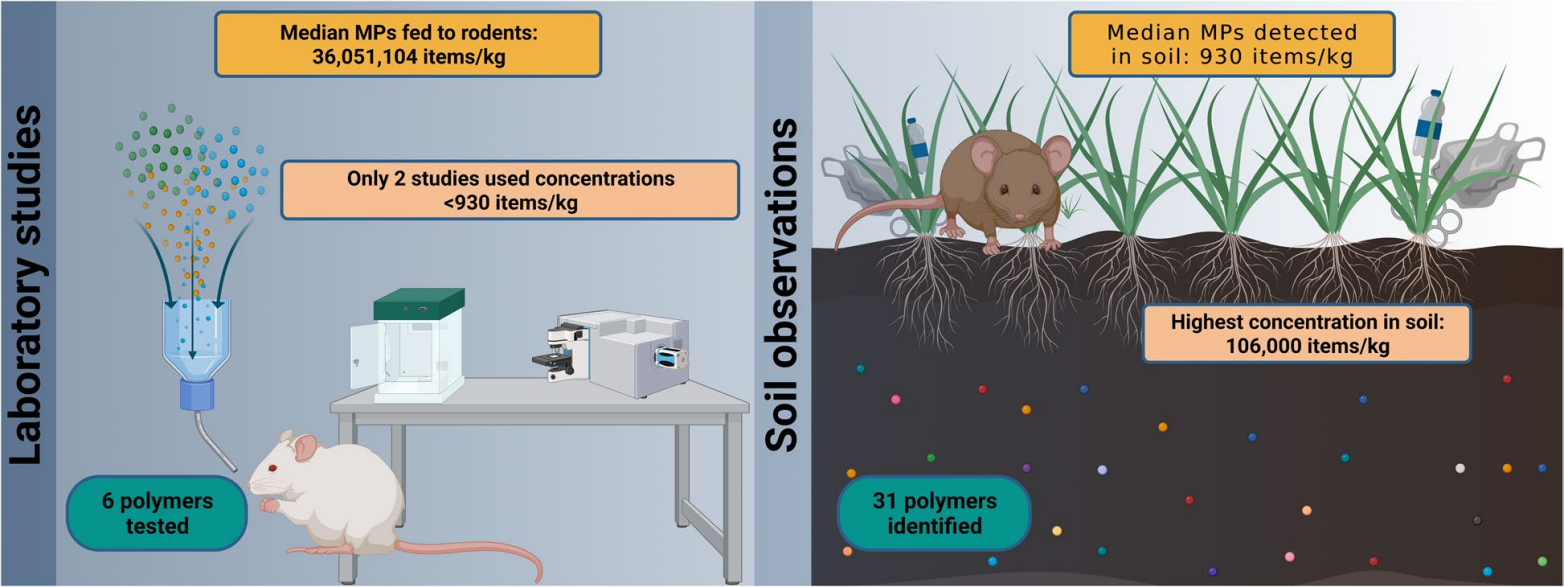

**Supplementary Information:**

The online version contains supplementary material available at 10.1186/s43591-023-00059-1.

## Introduction

The invention of plastics in the early 1900s revolutionized human societies [1], yet the excessive consumption of short-lived and single-use plastics has resulted in plastics accumulating almost everywhere on Earth [2, 3]. Plastic pollution is now so widespread that microplastics – plastic particles between 1 µm and 5 mm – are regularly detected in biological samples surveyed for their presence [4, 5]. Their resistance to degradation and ubiquitous nature make microplastics a worrying environmental contaminant, yet, despite their pervasiveness, very little is known about how microplastics might be impacting the health of species living in terrestrial ecosystems. This stands in stark contrast to the fact that 80% of species live on land [6], and that the volume of microplastics in terrestrial systems may be greater than that in oceans [7, 8].

Though evidence is still extremely limited, emerging studies are attempting to identify the predicted no-effect concentrations (PNEC) of soil microplastics for different taxa [9, 10]. For terrestrial vertebrates, studies carried out on rodents are showing that microplastics represent a potentially serious health threat, and may impact an array of biological functions [11, 12]. For instance, recent work in mice and rats has demonstrated the detrimental effects of microplastics on sperm production [13]. Similarly, a study conducted by Wang et al. [14] indicated that mice exposed to microplastics experienced both necroptosis and inflammation within bladder epithelium, while Djouina et al. [15] found that microplastics can adversely affect the small intestine and colon of mice, causing histological and immune disturbances, as well as inflammation. Data have been primarily limited to in vivo studies on laboratory rodents that were force fed plastics, however, and there are currently no studies describing the health effects of microplastics exposure on terrestrial vertebrates outside of laboratory settings. Thus, although the findings from these studies are certainly worrying, the extent to which they are representative of the conditions that humans and animals are actually experiencing in the real world is largely unknown. Here, we review the peer-reviewed literature to explore the extent to which lab studies on the effects of microplastics are representative of the conditions that terrestrial animals are experiencing in the real world. In particular we focused on understanding how the concentrations of microplastics and types of polymers being administered to rodents in lab studies compared to those found in terrestrial soils. Although our focus was on microplastics in soils, this is not the only path of exposure to microplastics. For instance, plants can uptake microplastics [16], which can then be ingested by herbivorous/omnivorous species. Airborne microplastics can also be inhaled, with intake rates that may be comparable to ingestion [17]. Most studies on airborne microplastics quantify concentrations in terms of deposition rates [18], however, making direct comparisons to lab studies impossible, and there is little information on the microplastic exposure and ingestion rates of free-ranging terrestrial vertebrates. Nonetheless, air and waterborne microplastics will ultimately accumulate in soils [18, 19], and soils are at the base of many terrestrial food webs [7]. The concentrations of microplastics in soils are thus likely to be broadly representative of exposure levels. Our results can help provide much needed context to the findings of existing health studies, as well as an ecologically relevant baseline that can help guide future lab studies on the health effects of terrestrial microplastics.

## Materials and methods

We first identified studies from the peer-reviewed literature that were focused on the health effects (i.e., changes in health resulting from exposure to a source) of microplastics on terrestrial rodents, or on microplastics in terrestrial soil environments. As our goal was to identify as many peer-reviewed articles as possible, we conducted a search for the terms “microplastics”, “microplastics” and “mice”, “microplastics” and “rats”, “microplastics” and “rodents”, “microplastics in lab”, and “microplastics in soil” on both Google Scholar and Scopus. We focused on microplastics in rodents as they are important model species for human and animal health research. Any in vivo lab studies not directly relating to the ingestion of microplastics were excluded as they were beyond the scope of our effort. Similarly, studies where soil samples were taken from lakes or river beds were excluded as our focus was on describing the conditions being experienced by terrestrial vertebrates. Through this initial search, 150 reviewed studies were compiled. For in vivo studies we extracted information on the polymer type, concentrations fed to laboratory rodents, and diameter, volume, and density of the microplastic particles. The microplastic type and final concentrations found in the soil environment were extracted from soil studies. There was very little consistency in the units across studies, and so to standardize microplastic measurements, all concentrations were converted to items/kg. To do this, polymer type was required to identify the density of the plastic, while diameter was required to calculate the volume. The known volume, density, and concentrations were then used in conjunction to calculate the number of particles and convert the data to items/kg. If any information required to make this conversion was absent from a study, it was excluded from subsequent analyses. Similarly, soil studies were excluded if information on the concentrations of microplastic were absent, or if they were experimental studies. This further narrowed the number of studies down to a total of 73 in vivo studies describing 183 experimental concentrations, and 41 soil studies with data on 93 sites. While it is possible that our literature search missed some relevant studies, the number of identified studies are in line with other recent reviews [20, 21] and our compiled dataset is thus likely to be representative of broad trends in the field. The full dataset is provided in Additional file 1 and the PRISMA checklist [22] in Additional file 2. The review process was not registered.

## Results and discussion

**Fig. 1:**
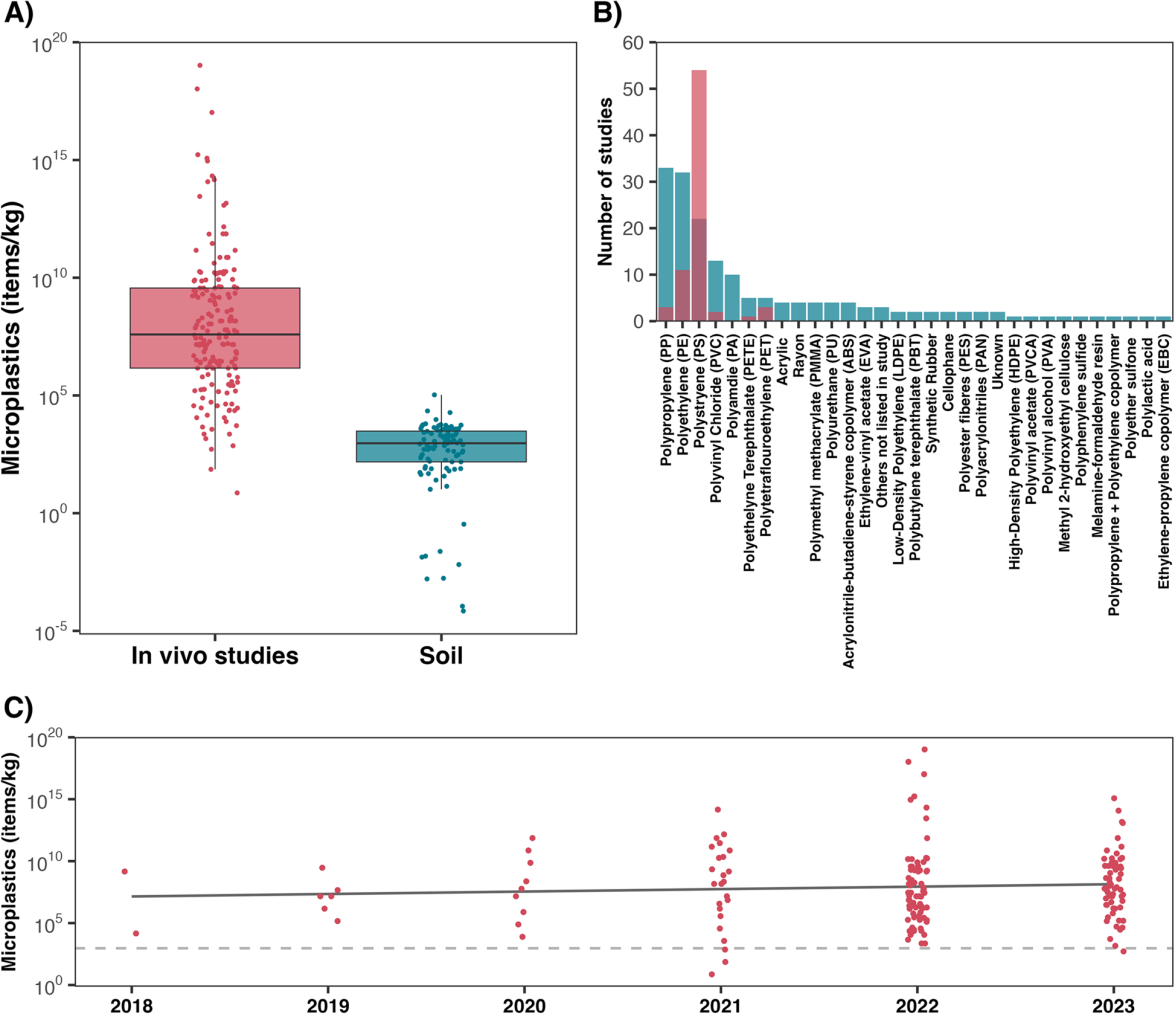
The boxplot in **A** shows the concentrations of microplastics fed to rodents in in vivo lab studies, compared to those of MPs found in soils. In **B** the number of soil studies which identified different plastic polymers are shown in blue, whereas the number of polymers assessed via in vivo health studies are shown in red. In **C**, the concentrations of microplastics being administered to rodents in in vivo studies is shown as a function of year of publication. The dashed line in **C** represents the median concentration of microplastics found in soils, while the solid line represents the trend of a fitted regression model. Data were compiled from 114 peer-reviewed studies; 41 on microplastics in soil and 73 on the health effects of microplastics

The median concentration of microplastics fed to laboratory rodents in in vivo studies was 39,051,103 items/kg. This was close to 42,000 times greater than the median concentration of 930 items/kg found in soil (Fig. 1A). Only two of the in vivo studies tested concentrations below this median value. The highest recorded mean concentration of microplastics in any soil sample was 106,000 items/kg which was found across various urban landscapes, including landfills, dumps, parking lots, industry and construction areas, urban parks, wetlands, forests, and agricultural areas in Coimbra, Portugal [23]; only 14 out of the 72 compiled lab studies used concentrations below this amount. We also found that while 31 different plastic polymers have been found to occur in soil, the health effects of only 6 polymers have been studied to date, with the overwhelming majority of in vivo experiments having focused on polystyrene (Fig. 1B). The stark contrast between the types and concentrations of microplastics being administered to lab rodents in in vivo studies versus the conditions these animals are likely to encounter in the wild illustrates the need for more ecologically realistic studies. Using a mixed effects regression model, we found that there was also no relationship between year of study and the concentrations of microplastics being administered to rodents in in vivo studies (β_Year_ = 0.25, 95% CI = -0.34–0.84; Fig. 1C). In other words, there was no evidence that studies are becoming increasingly environmentally realistic over time.

Notably, and in light of this disconnect, a common trend across lab studies was the lack of any rationale for the concentrations of microplastic that were administered. The 31 studies that did provide justification chose concentrations that were based either on the concentrations of microplastic found in rivers [24–26], or on existing in vivo studies [12, 14, 27–51]. For instance, Yang et al. [34] and Mu et al. [30], both based their study designs on work on mice by Deng et al. [52]. Deng et al. [52], however, based their study on microplastics concentrations found in rivers, and therefore it does not accurately reflect exposure levels in terrestrial environments. In addition, while studies often use high concentrations of potential toxicants to establish dose-effect relationships, it is rare for acute or chronic ratios to exceed 100-fold greater than environmentally relevant concentrations [53]. In other words, the 4 order of magnitude difference between the microplastics administered in in vivo health studies and environmental concentrations can not be attributed to standard practice in dose-effect research. It is likely that the lack of any rationale contributed to the high variability in concentrations used between studies. Thus, while a handful of lab studies did provide some form of justification for their study design, the extent to which these studies are representative of the conditions that humans and animals are actually experiencing in the real world is limited.

## Conclusions

Plastic pollution is arguably one of the most pressing ecological and public health issues of our time, yet existing research on the health effects of terrestrial microplastics does not accurately reflect the conditions that humans and animals are actually experiencing. This is in line with earlier findings from toxicology work on microplastics in aquatic environments [54–56], and our analyses showed no indication that exposure concentrations are becoming more realistic over time (Fig. 1C). Paired with this disconnect is the fact that 3,067 animals were sacrificed to generate the findings of these 73 studies, yet the majority of these animals were fed tens to hundreds of thousands of times more plastic than they would ever be exposed to in the wild. Because microplastics research also receives frequent media attention, performing true-to-life studies is of the utmost importance so as to not erode the public’s faith in the scientific process. It therefore falls on the scientific community to describe the ecologically realistic effects of microplastics on the health of terrestrial species in order for well-founded mitigation efforts to be launched. 

## Supplementary Information

**Additional file 1. **Compiled microplastics dataset.

**Additional file 2.** PRISMA 2020 Main Checklist.

## Data Availability

The data and R scripts used to carry out this study are openly available on GitHub at https://github.com/QuantitativeEcologyLab/MP_Disconnect.
